# The fitness effects of a pale mutant in the aposematic seed bug *Lygaeus simulans* indicate pleiotropy between warning coloration and life history

**DOI:** 10.1002/ece3.4723

**Published:** 2018-12-04

**Authors:** Vicki L. Balfour, Cédric Aumont, Liam R. Dougherty, David M. Shuker

**Affiliations:** ^1^ School of Biology University of St Andrews St Andrews UK; ^2^ Agrocampus‐Ouest Rennes France; ^3^ Department of Evolution, Ecology and Behaviour University of Liverpool Liverpool UK

**Keywords:** aposematism, color polymorphism, life‐history, pleiotropy, supergene

## Abstract

Conspicuous warning colors that signal chemical or other defenses are common in the natural world. For instance, such aposematic warning patterns of red‐and‐black or yellow‐and‐black are common among insect taxa, particularly in the order Hemiptera, often forming the basis of Batesian and/or Müllerian mimicry rings. In addition, it has been repeatedly noted that color polymorphisms or mutants that influence pigmentation can show pleiotropy with other behavioral, physiological, or life‐history traits. Here, we describe a pale mutant of the seed bug *Lygaeus simulans* that appeared in our laboratory population in 2012, which differs in color to the wild‐type bugs. Through multigenerational experimental crosses between wild‐type and pale mutant *L. simulans*, we first show that the pale phenotype segregates as a single Mendelian locus, with the pale allele being recessive to the wild type. Next, we show (a) that there is a large heterozygous advantage in terms of fecundity, (b) that pale females suffer reduced longevity, and (c) that pale males have increased body length compared to wild‐type homozygotes. Our data therefore suggest that the color locus is pleiotropic with a number of life‐history traits, opening the door for a more complete genetic analysis of aposematic coloration in this species. In addition, this phenotype will be useful as a visible genetic marker, providing a tool for investigating sperm competition and other post‐copulatory drivers of sexual selection in this species.

## 1 INTRODUCTION

Conspicuous warning colors that signal chemical or other defenses are common in the natural world (aposematism: Cott, 1940; Rojas, Valkonen, & Nokelainen, 2015a; Ruxton, Sherratt, & Speed, 2004). Among insects, aposematic patterns of red‐and‐black and yellow‐and‐black are widespread taxonomically, indeed forming the basis of inter‐connected Batesian and Müllerian mimicry rings (Ruxton et al., 2004; Sherratt, 2008). The evolution of aposematic colors has long been of interest (Guilford, 1990; Lindström, 1999; Mappes, Marples, & Endler, 2005; Poulton, 1890; Rowe & Guilford, 2000; Wallace, 1867), as has been the evolution of mimicry of aposematic species (Ruxton et al., 2004). In terms of coloration, it has been repeatedly noted that color polymorphisms or mutations that influence pigmentation can show pleiotropy with other behavioral, physiological, or life‐history traits (McKinnon & Pierotti, 2010; Rojas, Gordon, & Mappes, 2015b; Sinervo & Svensson, 2002; Wittkopp & Beldade, 2009). These patterns suggest shared genetic pathways, either through a gene directly influencing both coloration and another phenotype, or through closely linked genes that influence one phenotype or the other (McKinnon & Pierotti, 2010; Wittkopp & Beldade, 2009). Indeed, recent work has reignited interest in so‐called “supergenes,” which are blocks of genes involved in regulating color, pattern, and other traits, and that are associated with chromosome inversions that reduces recombination between loci in the supergene (e.g., wing patterning in *Heliconius* butterflies: Joron et al, 2006; 2011; Jones & Salazar, 2012; Kronforst & Papa, 2015; and *Papilio* butterflies: Clarke & Sheppard, 1960; Kunte et al, 2014; color morphs in ladybirds: Marples, Jong, Ottenheim, Verhoog, & Brakefield, 1993; shell color and patterning in snails: Murray & Clarke, 1976a; 1976b; social behavior in fire ants (Ross & Keller, 1998): reviewed by Thompson & Jiggins, 2014).

**Figure 1:**
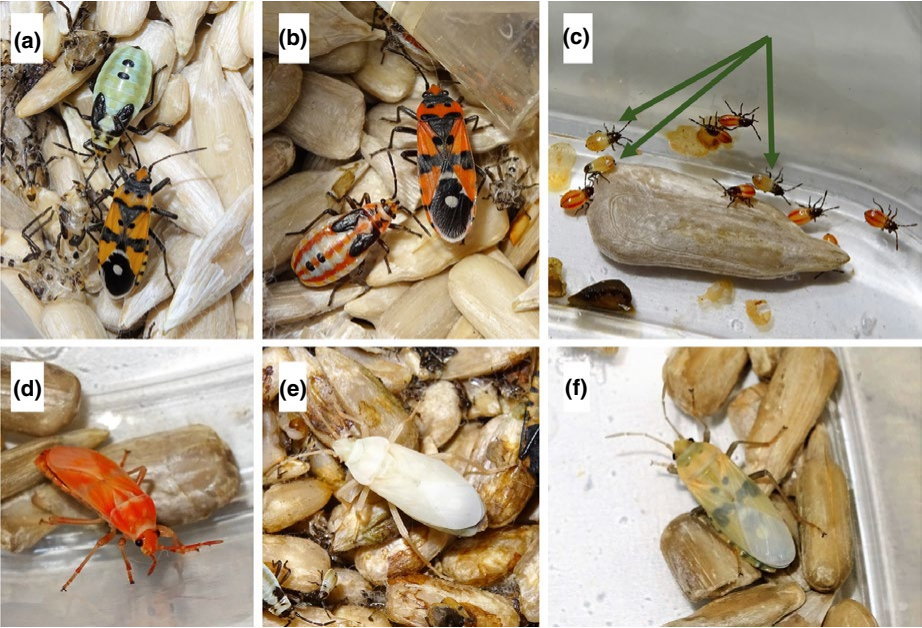
The pale and wild‐type morphs of *Lygaeus simulans*. (a) Below: adult pale morph; above: fifth‐instar pale morph. (b) Right: adult wild‐type morph; left: fifth‐instar wild‐type morph. (c) Mixture of pale (green; arrows) and wild‐type (red) first‐instar nymphs. (d) Newly eclosed adult wild‐type morph. (e) Initial color of newly eclosed adult pale morph. (f) Faded color of recently eclosed adult pale morph. All bugs were raised on *Helianthus annuus* sunflower seeds throughout. *Photo credits: Vicki Balfour*

One insect order in which warning coloration is fairly common is the Hemiptera, or true bugs. For instance, in the Lygaeidae, which includes the seed bugs, a sample of 1951 species from a national museum collection contained more than 20% species that were aposematic, with approximately 14% of 452 genera containing at least one aposematic species (Burdfield‐Steel & Shuker, 2014; taxonomically, the traditional family Lygaeidae is now known to be polyphyletic, subsumed within the super‐family Lygaeiodea, which contains the seed and milkweed bugs, stilt bugs, and ground bugs: Weirauch & Schuh, 2011). These colors, often red‐and‐black (Figure 1b), warn predators that these species are chemically defended, typically by cardiac glycosides obtained from their food plants (Aldrich et al., 1997; Aldrich, 1988; Mappes et al., 2005; Scudder & Duffey, 1972;Sillén‐Tullberg, 1985a; reviewed by Burdfield‐Steel & Shuker, 2014).

Here, we consider the seed bug *Lygaeus simulans*, which displays classic red‐and‐black warning coloration (hereafter referred to as “wild‐type” coloration). *Lygaeus simulans* is the sibling species to *L*. *equestris* and indeed has only been described relatively recently (Deckart, 1985). The two species are differentiated by antennal and genital morphologies, but are otherwise indistinguishable by eye, and do hybridize in the laboratory (Evans, Nowlan, & Shuker, 2015). Given this difficulty, species distributions are probably tentative, but *L*. *equestris* is considered to be broadly distributed across Europe, reaching Sicily, Spain, and Sweden, while *L*. *simulans* is parapatric with *L. equestris* in central and southern Europe, with our population of *L*. *simulans* coming from Tuscany, Italy.

During routine laboratory culture, we noticed the appearance of a pale mutant of *L*. *simulans* in 2012 (we note that a “gray” mutant was observed in a laboratory population of *L*. *equestris* from northern Italy, with mutant fifth‐instar nymphs being described as “gray” in coloration, and there may be similarities between the two: Sillén‐Tullberg, 1985a; 1985b). Both *L*. *equestris* and *L*. *simulans* have recently been the focus of a range of studies exploring the reproductive behavior and ecology of the species, including work on heterospecific matings (reproductive interference: Shuker, Currie, Hoole, & Burdfield‐steel, 2015), mating failure (Greenway & Shuker, 2015; Greenway, Balfour, & Shuker, 2017), and pre‐ and post‐copulatory sexual selection (Burdfield‐Steel , Auty, & Shuker, 2015; Dougherty & Shuker, 2014, 2016; Dougherty, Rahman, Burdfield‐Steel, Greenway, & Shuker, 2015). The presence of a visible genetic marker would facilitate a number of experimental techniques, especially in terms of understanding patterns of intra‐ and inter‐specific sperm precedence. However, the nature of the mutant also speaks more generally to the question of the genetic basis of aposematic colors and patterns. As such, here we report (a) the patterns of inheritance of the pale mutant, showing that it is a recessive mutation that inherits as a single Mendelian locus; (b) analysis of the fitness—in terms of fecundity and survival—of different genotypes, comparing wild‐type and mutant homozygotes and their heterozygotes. Our data suggest that the color locus is pleiotropic with some life‐history traits, opening the door for a more complete genetic analysis of aposematic coloration in this species.

## 2 MATERIALS AND METHODS

### 2.1 Study organism and description of the pale mutant

*Lygaeus simulans* were collected by DMS from the Pratomagno Hills in Tuscany, Italy, in 2008 and 2009, and transferred to the Shuker laboratory at the University of St Andrews in 2009. In the laboratory, we kept the bugs in continuous culture population cages (30 × 15 × 15 cm plastic boxes) with an ad libitum supply of dehusked, organic sunflower seeds, cotton wool for habitat, and two cotton‐plugged tubes of distilled water (25 ml) which are changed once a week. The bugs were housed in an incubator at 29^O^C with a 22:2 hr light: dark cycle to prevent the onset of reproductive diapause. A minimum of two replicates of the population cages are kept at any one time. We created new population cages approximately every 6–8 weeks by transferring around 50 bugs from across each instar category (i.e., nymphs to adults) from at least two separate population cages into a new cage. This was to maintain gene flow and limit inbreeding.

Following their first appearance in the Shuker laboratory in 2012, a population cage of pale *L. simulans* was created in 2013 by LRD by removing all pale individuals from the wild‐type *L*. *simulans* population boxes when they appeared, and also from an F3 generation from a breeding experiment, also carried out by LRD (unpublished data). This population was maintained under the same conditions as the wild‐type population.

**Figure A1:**
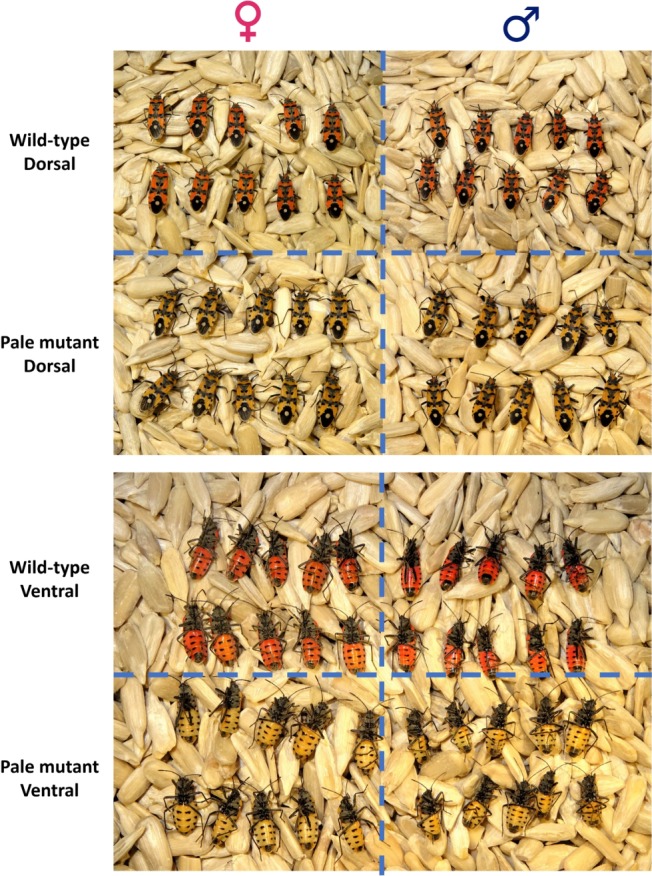
Comparison of female (left) and male (right) wild‐type and pale mutant *L. simulans *from the dorsal (top) and ventral (bottom) view. Bugs aged 15–16 days post‐eclosion were placed in the freezer for 15 mins to immobilize them so that they would remain still for photographs. This did not kill the bugs. 10 females and 10 males of each color morph were photographed. Note: The top four panels are not to exact scale with one another, whereas the bottom four are. *Photo credits: Vicki Balfour*

Other than color (Figure 1a), pale *L*. *simulans *individuals appear to be almost identical to the wild‐type individuals (Figure 1b; see also Figure A1 in the Appendix) in every way, the exception being that the pale mutant individuals appear—anecdotally—to be more active and more prone to try and escape from population cages. However, activity and escape responses are not phenotypes that we consider here (see below). Although adults are a pale brown‐green color instead of red‐orange, they share the same markings on their body and wings and appear to undertake similar behaviors. All nymphal instars are likewise green instead of orange, though they again share the same markings as their wild‐type counterparts (Figure 1c). Wild‐type eggs are white when first oviposited, then after 2–6 days they turn bright red if they are fertile and about to hatch. Pale morph eggs on the other hand take on a greenish tinge when they are about to hatch, but this color change is more subtle and less easy to observe. This confirms that the influence of the color mutation begins to act before hatching. When wild types first eclose into adults, they are a bright luminous orange (Figure 1d) which fades to the aposematic black and orange over the next couple of hours (Figure 1b). When pale morphs first eclose into adults however, they are almost white (Figure 1e) and this fades to a light brown, the same color of sunflower seeds (Figure 1f), before eventually becoming the green‐brown color after a couple of hours (Figure 1a).

We do not know for certain what controls pigmentation in *L. simulans*. However, in another Lygaeidae species, *Oncopeltus fasciatus*, the black pigmentation is produced via the melanin pathway, with Dopamine melanin being the primary component (Liu, Lemonds, & Popadić, 2014). Liu et al's (2014) results also indicated that this pathway might have a slight involvement in orange pigmentation production in *O. fasciatus*. Otherwise, evidence points toward pteridines being the main component of orange pigmentation in this species, and indeed other Hemiptera, with isoxanthopterin and xanthopterin being found in all body tissues (Forrest, Menaker, & Alexander, 1966; Hudson, Bartel, & Craig, 1959). Therefore, we might expect similar pigments and pathways to be present in *L. simulans*.

### 2.2 Experiment 1. Testing mendelian inheritance

To obtain virgin bugs for the experimental crosses, we collected late‐instar nymphs from wild‐type (W) or pale (P) population cages and transferred them, using an aspirator, to nymph boxes (20 × 10 × 8 cm plastic boxes), supplying them with a cotton‐plugged water tube (25 ml), an ad libitum supply of sunflower seeds, and a piece of cotton wool for habitat. We checked nymph boxes every 2–3 days for newly eclosed adults, which we then separated by sex into same‐sex tubs (108 × 82 × 55 mm plastic deli tubs), with a maximum of 10 individuals per tub. We provided each tub with an ad libitum supply of sunflower seeds, a cotton‐plugged water tube (7 ml), and a piece of cotton wool for habitat. This was to ensure that all bugs were virgins, as *L. simulans* do not become sexually mature until around 7 days post‐eclosion.

We made four sets of experimental crosses: PxP, PxW, WxP, and WxW (first letter denoting the female's phenotype, the second the male's phenotype, P denotes the pale mutant phenotype, W the wild type). The sample sizes for each treatment were *N* = 121, 122, 120, and 123, respectively. Offspring were obtained from *N* = 106, 102, 96, and 91 pairs, respectively. For each cross, we paired virgin males and females (7–14 days post‐eclosion) in individual tubs (108 × 82 × 55 mm) and provided them with a cotton‐plugged water tube (7 ml) and 20–30 sunflowers seeds. We returned pairs to the incubator and left them to mate for 3 days (to limit mating failure: Greenway & Shuker, 2015). After 3 days, we separated the pairs and euthanized the males by putting them in the freezer at −18°C. We then left females in the same tub and returned them to the incubator to allow them to lay eggs for a further 7 days. We then removed the females and similarly euthanized them, before scoring tubs for the presence/absence of eggs. If eggs were present, we returned the tubs to the incubator for a further 7 days. We discarded any tubs which had no eggs. After 7 days, we scored tubs for the presence/absence of nymphs and for the presence/absence of each color morph of nymph. If only one color morph of nymph was present (e.g., wild type), then we did not count the nymphs and just recorded the morph present. If both color morphs were present, we froze the tub at −18°C for a minimum of 24 hr and then counted every individual nymph of each color morph. Freezing live material does not influence coloration, and hence scoring of phenotype, and makes subsequent counting easier. These individuals were also not needed for subsequent crosses (as we did not know the genotype of wild‐type individuals).

To generate the F2 offspring, we used nymphs from tubs that produced only one morph of nymph. We transferred a maximum of 10 F1 nymphs (randomly selected) from each tub to a nymph box (20 × 10 × 8 cm) with other F1 nymphs from the same treatment and supplied them with a cotton‐plugged water tube (25 ml), and ad libitum supply of sunflower seeds and a piece of cotton wool for habitat as before. Only nymphs from the same treatments were kept in the same nymph box. There were six or seven nymph boxes per treatment (for treatments PP and WW: *N* = 7; for treatments PW and WP: *N* = 6; total *N* = 26). We then reared the F1 nymphs to adulthood, checking the nymph tubs boxes every 2–3 days for newly eclosed adults as before. We separated newly eclosed adults by sex into same‐sex tubs (108 × 82 × 55 mm) containing a maximum of 10 individuals (from the same treatment) and provided then with a cotton‐plugged water tube (7 ml), an ad libitum supply of sunflower seeds, and a piece of cotton wool for habitat.

At 7–14 days post‐eclosion, we paired up virgin males and females with other F1 individuals of the opposite sex (from the same treatment) in individual tubs (108 × 82 × 55 mm) and again provided them with a cotton‐plugged water tube (7 ml) and 20–30 sunflowers seeds. Sample sizes were as follows: PP: *N* = 93, PW: *N* = 119, WP: *N* = 120, and WW: *N* = 119, with treatment codes denoting the phenotype of the mother (first letter) and the father (second letter) of the F1 generation. We kept pairs in the incubator and allowed them to mate for 3 days, before removing the males and allowing the females to oviposit for 7 days prior to checking for eggs. We then removed the females and left the eggs in the incubator for 7 days before checking for nymphs as before. Boxes were then frozen and nymphs scored for color and number. Offspring were obtained from *N* = 73, 109, 109, and 93 pairs, respectively. For some boxes, it was not possible to accurately identify nymphs (due to some nymphs dying before they were frozen, which can alter their color) so these were excluded from the analysis; therefore, the analyzed sample sizes were *N* = 73, 101, 103, and 93, respectively.

### 2.3 Experiment 2: Fitness comparisons between the color morphs

To obtain adults for this experiment, we checked the F1 nymph boxes from Experiment 1 every day for the presence of adults and transferred them to same‐sex tubs as before. These tubs were again provisioned with an ad libitum supply of sunflower seeds and a cotton‐plugged water tube (7 ml). Adults were not mixed with individuals from other treatments or nymph boxes. Therefore, for the following experiment, we noted which nymph box individuals had been raised in (there were three nymph boxes per treatment that adults were extracted from) and the tub density (ranging from 1 to 10 bugs) that individuals were kept in as adults between 1 and 7 days post‐eclosion.

At 7 days post‐eclosion, we paired up males and females from the same treatment (pairs per treatment: PP: *N* = 47; PW: *N* = 50; WP: *N* = 50; WW: *N* = 50; once again, treatment codes denote the phenotype of the mother (first letter) and the father (second letter) of the F1 generation) and allowed them to mate for 3 days in a tub (108 × 82 × 55 mm) with 10–15 sunflower seeds and a cotton‐plugged water tube (7 ml). To facilitate the attainment of similar sample sizes, seven males in the PP treatment were 8 days old when paired up. We checked the pairs twice daily to see whether they were mating or not (seven checks in total). Copulations in *Lygaeus* can typically last several hours and include contact post‐copulatory mate guarding, so we are likely to sample many of the copulations that occur (Micholitsch, Krügel, & Pass, 2000; Shuker, Ballantyne, & Wedell, 2006; Sillén‐Tullberg, 1981). Following this, we separated the pairs, leaving females in the same tub while transferring males to new individual tubs (108 × 82 × 55 mm) with 10–15 sunflower seeds and a cotton‐plugged water tube (7 ml).

We checked all bugs for survival daily, and if any bugs were found dead, we recorded the date of death and age in days and then froze the bug at −18°C, for later measurements. If a female died during the three‐day mating phase, we checked the tub for the presence of eggs and transferred the male to an individual tub. If there were no eggs present, then we discarded the original tub. If eggs were present, we counted them, then returned them to the incubator for a further 7 days. After 7 days, we froze the tubs and counted any nymphs that were present. If a male died during the mating phase, then we left the female in the original tub and carried on the experiment as if the mating phase was finished, monitoring the female for survival and the presence of eggs as described below.

We checked females every 3–5 days for the presence of eggs, and if eggs were present, we transferred females to a new tub, counting the eggs present in the previous tub, before returning them to the incubator for a further 7 days. After 7 days, we froze the tubs and counted the total number of nymphs and noted whether wild‐type, pale, or both nymph morphs were present. We only counted wild‐type and pale nymphs separately if they arose in a treatment they were not supposed to (i.e., pale morph nymphs in the treatment WW or wild‐type nymphs in the treatment PP). This did occur (see below for details) and could have been due to scoring error (though nymphs were checked very carefully) or due to the accidental transfer of eggs from one tub to another. To count the eggs, we had to separate egg clumps using forceps, and although we wiped forceps between tubs, there is the possibility that some eggs remained on the forceps and were transferred to other tubs.

We transferred males to a fresh tub once every 2 weeks as we tracked their longevity, and all bugs were kept for a maximum of 8 weeks (9 weeks post‐eclosion) or until death.

We (VLB) measured the body length of all the bugs after thawing using a dissecting microscope fitted with an eyepiece micrometer. We measured the length from the tip of the snout to the tip of the wings, dorsal side up. We remeasured 64 bugs (32 males and 32 females), blind to the original measurements, to check measurement reliability. Our measurements were highly repeatable (intraclass correlation coefficient: *r* = 0.973; one‐way ANOVA: *F*_63,64_ = 75.53, *p* < 0.001; Lessells & Boag, 1987).

### 2.4 Analysis

We carried out all analyses using R statistical software (R Core Team, 2015). For Experiment 1, a one‐sample *Z* test was used to test whether the observed F2 offspring ratios of wild‐type: pale nymphs differed from the expected ratio of 3:1 for a trait inherited in a Mendelian fashion. For Experiment 2, general linear models (GLMs) were used to test the effect of treatment on fecundity and body size. General linear models were also used to look at the relationship between the number of eggs laid and the number of nymphs produced, and also the effect of the number of times a pair were observed mating, the effect of body size, and the effect of age on fecundity. ANCOVAs were used to test interactions between treatment and age, or number of times observed mating, on the number of eggs and nymphs produced, or between treatment and number of eggs laid on the number of nymphs produced. Log‐rank tests were used to test for any survival differences between treatments for both males and females.

## 3 RESULTS

### 3.1 Experiment 1. Testing mendelian inheritance

**Figure 2:**
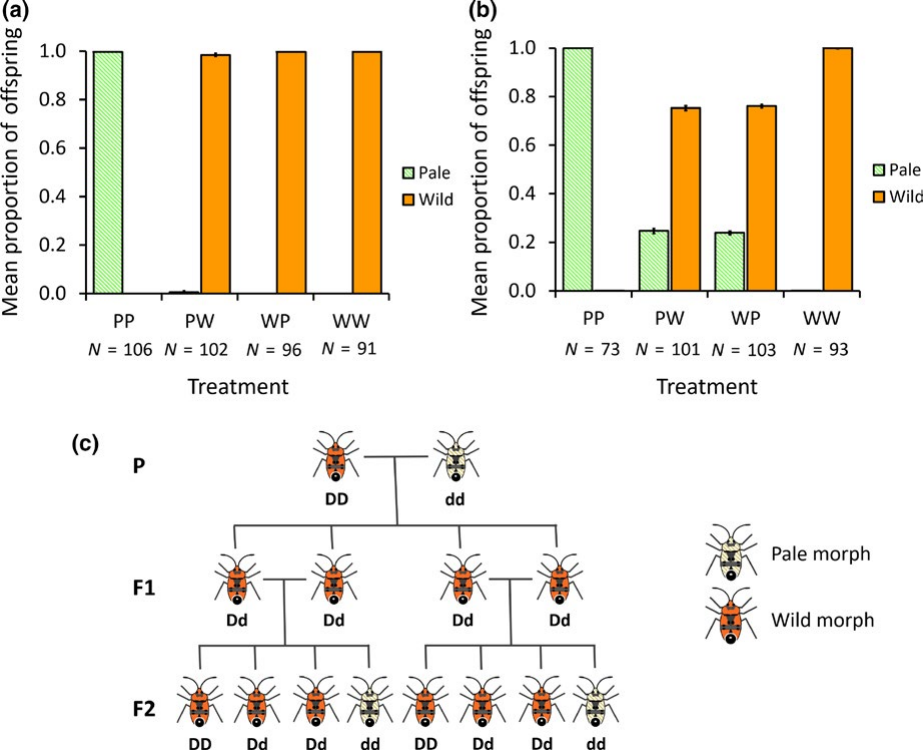
(a) The proportion of each nymph morph in F1 generation produced by the four experimental cross treatments. The proportion of wild‐type offspring are shown in orange and the pale offspring shown in hatched green. Error bars are binomial standard errors. Treatments are as follows: PP = pale female x pale male; PW = pale female × wild‐type male; WP = wild‐type male × pale female; WW = wild‐type female × wild‐type male. Sample sizes for each treatment are shown below the treatment abbreviation. Only in the treatment PW, did one pair produce both wild and pale morph nymphs. (b) The proportion of each nymph morph produced in the F2 generation produced by the four experimental crosses. Other details are as above. (c) The expected results from a Parental (P) cross between a homozygous wild‐type (denoted here as DD for dominant allele) and a homozygous recessive pale individual in the F1 and F2 generations

The pale visible mutant in *L*. *simulans* is inherited as a single locus in a strictly Mendelian fashion, with the pale mutant allele being recessive (see Figure 2c for pattern of inheritance). The data for the F1 are shown in Figure 2a. WW and PP crosses produced only wild‐type or pale phenotypes, respectively. In the WP cross, there were only wild‐type offspring produced, while in the PW cross we observed only wild‐type offspring from 101 of the 102 crosses that produced nymphs, with 10 wild‐type nymphs and 13 pale nymphs being produced in the other cross. This ratio of offspring suggests that the wild‐type father in this cross was a rare heterozygote in the stock population and that the mutant is still segregating in that population, albeit at a very low frequency.

In the F2, for the treatment PW, in which both individuals in the pair were heterozygous, the 3:1 wild‐type: pale offspring phenotype ratios expected under Mendelian segregation with a dominant wild‐type allele were produced (PW: proportion wild type = 0.753 ± 0.005; $\chi_{1}^{2}$ = 0.37, *p* = 0.543, C.I. = 0.744–0.762). For the other heterozygote cross (WP), there was a very small deviation from 0.75, suggesting a very slight bias toward the production of wild‐type offspring (WP: proportion wild type = 0.761 ± 0.005; $\chi_{1}^{2}$ = 6.06, *p* = 0.014, C.I. = 0.752–0.770; Figure 2b). However, the effect is very small across the 8,800 offspring scored for this treatment, and we consider the inheritance effectively Mendelian.

In total, 28,726 offspring were scored for color morph during this experiment. The mean number of offspring produced by all the crosses is given in Table 1. In the treatment PP, one of the 73 pairs that produced offspring produced two wild‐type nymphs, while in the treatment WW, one of the 93 pairs that produced offspring produced one pale nymph. In each case, we cannot exclude experimental error, such as mis‐scoring the phenotype or accidentally misplacing a nymph during counting. However, there is also the small possibility that we have caught mutations to either a wild‐type‐like allele in the first case, or to the pale form in the latter. All other offspring in these crosses were pale or wild type, respectively, as expected.

**Table 1:** Descriptive statistics for Experiment 1 in terms of the mean number of nymphs produced by the four F1 experimental cross treatments

Experimental cross	*N*	Number of nymphs								
		Wild			Pale			Total		
		Mean	*SE*	Range	Mean	*SE*	Range	Mean	*SE*	Range
PP	73	0.03	0.03	0–2	59.5	4.5	1–223	59.6	4.5	1–223
PW	101	64.6	2.4	0–120	21.2	1.0	0–45	85.8	3.1	1–165
WP	103	65.0	2.5	1–125	20.4	0.9	0–40	85.4	3.2	2–158
WW	93	74.3	3.9	1–155	0.01	0.01	0–1	74.4	3.9	1–155

### 3.2 Experiment 2: Fitness comparisons between the color morphs

Out of 194 pairs, 189 laid eggs (97.4%) and 175 produced nymphs (90.2%; one female escaped (treatment = PP) and two females were killed (treatment = PW) during the experiment, and the data discarded). Therefore, the mating failure rate was 9.8%. This is low compared to previous studies on *L. simulans*, showing that giving the bugs the opportunity to mate multiple times with the same partner over 3 days reduces failure to transfer or receive sperm.

In total, there were 92,188 eggs and 55,456 nymphs counted during this experiment. All homozygous wild‐type pairs (treatment = WW) which produced nymphs had exclusively wild‐type nymphs (*N* = 44) except for one pair which had one pale nymph in among all its wild‐type nymphs. See methods above for possible explanations for this. All heterozygous pairs produced both wild‐type and pale nymphs (*N* = 92), except for one pair in each treatment. The pair in the treatment PW only produced two wild‐type nymphs over the 8 weeks, while the pair in the WP treatment only produced three wild‐type nymphs. Most pale homozygous pairs (treatment = PP) produced exclusively pale nymphs (*N* = 22); however, some pairs (*N* = 14) had small numbers (<8) of wild‐type nymphs among their pale nymphs. In total, there were 33 wild‐type nymphs out of the 6,597 nymphs counted in the pale treatments, making 0.5% of the offspring “wrong.”

**Figure 3:**
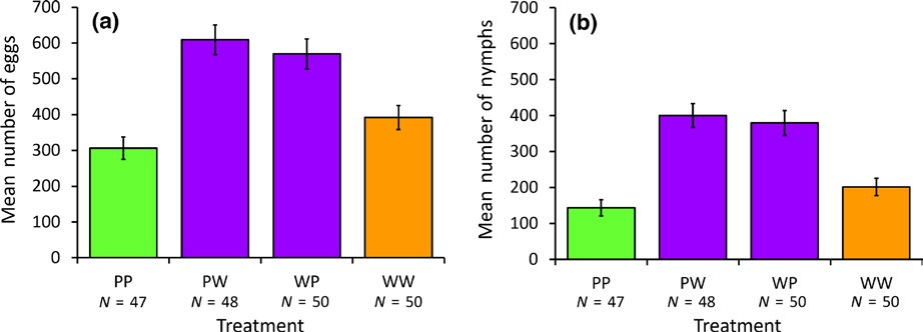
Comparison of (a) number of eggs and (b) number of nymphs produced by females in the four different experimental cross treatments. Error bars are the standard error. Homozygous pale morphs (PP) are shown in green, heterozygous wild‐type morphs (PW and WP) in purple, and homozygous wild‐type morphs (WW) in orange. The sample sizes of the number of pairs are indicated below the treatment codes

There was a significant effect of genotype on offspring production. Fecundity differed significantly between treatments, both in terms of the number of eggs produced (GLM: *F*_3,190_ = 14.36, *p* < 0.001; Figure 3a) and in terms of the number of nymphs produced (*F*_3,190_ = 18.89, *p* < 0.001; Figure 3b). In both cases, there is clear evidence of heterozygote advantage, with heterozygotes producing more eggs and nymphs on average compared to homozygotes (comparing homozygotes and heterozygotes: number of eggs laid: *F*_1,192_ = 39.75, *p* < 0.001; number of nymphs produced: *F*_1,192_ = 54.41, *p* < 0.001; for descriptive data, see Table 2).

**Table 2:** Descriptive statistics for Experiment 2 in terms of the mean number of eggs and nymphs produced by the four experimental genotypes

Genotype	*N*	Number of eggs			Number of nymphs		
		Mean	*SE*	Range	Mean	*SE*	Range
PP	46	306.4	30.9	0–739	143.4	22.2	0–493
PW	48	609.0	42.0	0–1,189	400.1	33.2	0–845
WP	50	569.4	42.0	13–1,065	379.5	34.5	0–804
WW	50	391.9	33.6	0–816	201.6	24.3	0–558

**Figure 4:**
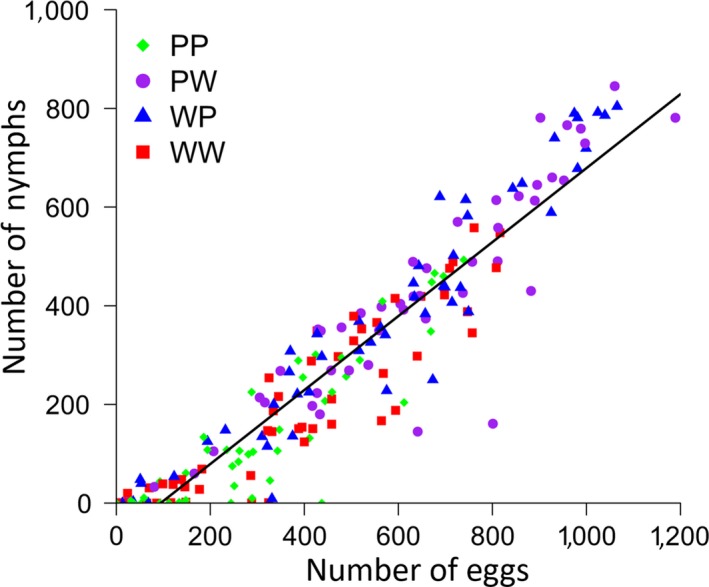
The relationship between the number of eggs and the number of nymphs produced by females (*N* = 194) across the four experimental cross treatments. A linear regression line of best fit is shown (Nymphs = 0.75*Eggs − 70.34)

Unsurprisingly, there was a strong positive correlation between the number of eggs laid and the number of nymphs produced (Pearson's correlation coefficient: *r*_192_ = 0.94, *p* < 0.001; *r* ^2^ = 0.88; Figure 4). This did not differ across treatments (ANCOVA: number of eggs: *F*_1,186_ = 970.48, *p* < 0.001; treatment: *F*_3,186_ = 0.18, *p* = 0.908; interaction: *F*_3,186_ = 2.22, *p* = 0.087).

**Figure 5:**
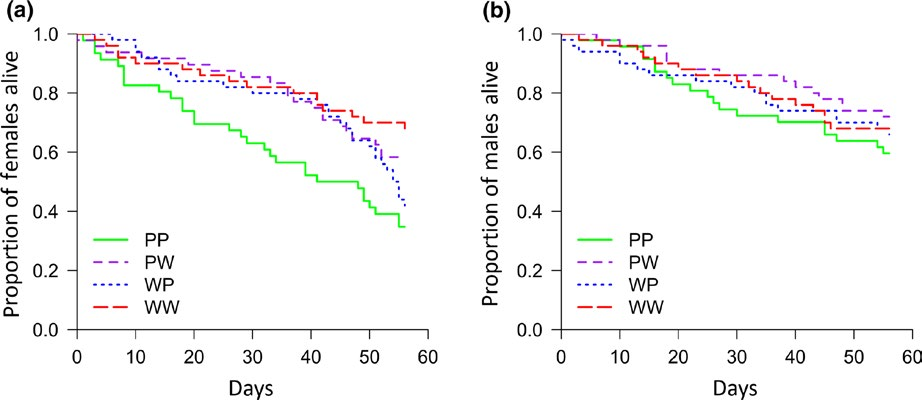
Survival curves for (a) females and (b) males during the 56‐day Experiment 2. Day 0 denotes the day that pairs were first paired up to mate (this is 7 days post‐eclosion). Males tended to live longer than females, and genotype only had a significant effect on female survivorship

In terms of longevity, there was much less of an effect of experimental cross and hence genotype. There was a significant difference in survivorship between females from the different treatments (log‐rank test: $\chi_{3}^{2}$ = 13.4, *p* = 0.003), with pale homozygotes suffering lower survival compared to the heterozygotes and the wild‐type homozygote (Figure 5a). However, there were no differences between males of the different crosses in terms of longevity ($\chi_{3}^{2}$ = 1.9, *p* = 0.59; Figure 5b). More generally, males lived significantly longer than females ($\chi_{1}^{2}$ = 8.2, *p* = 0.004; Figure 5a,b).

Females that lived for longer laid more eggs (GLM: *F*_1,192_ = 51.23, *p* < 0.001) and produced more nymphs (*F*_1,192_ = 23.64, *p* < 0.001) than females which died sooner. This is expected because females which lived for longer had more time to lay eggs. This relationship did not differ across treatments (ANCOVA: female age: *F*_1,186_ = 51.14, *p* < 0.001; treatment: *F*_3,186_ = 0.08, *p* = 0.971; interaction: *F*_3,186_ = 1.93, *p* = 0.127). Interestingly, males which lived for longer were also paired with females which laid more eggs (GLM: *F*_1,192_ = 7.92, *p* = 0.005) and they sired more offspring (*F*_1,192_ = 8.14, *p* = 0.005) than males which died sooner. Although significant, this relationship is not as strong as for females and is most likely driven by the few males that died during the mating phase and hence did not get to mate as often as males which survived the whole mating phase.

**Figure 6:**
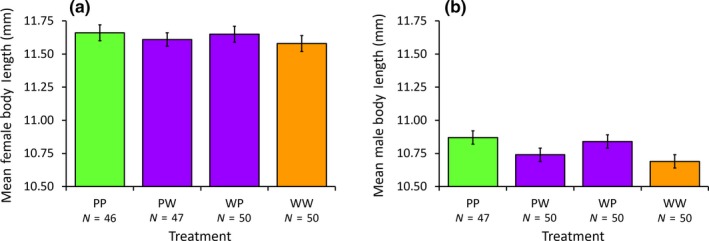
Comparison of (a) female body length and (b) male body length between the four experimental cross treatments. Error bars are the standard error. Homozygous pale morphs (PP) are shown in green, heterozygous wild‐type morphs (PW and WP) in purple, and homozygous wild‐type morphs (WW) in orange. The sample sizes of the number of individuals measured are indicated below the treatment codes

In terms of body size, there was no effect of genotype on female body length (*F*_3,189_ = 0.41, *p* = 0.75; Figure 6a). However, male body length did differ depending on genotype (*F*_3,193_ = 2.73, *p* = 0.045), with homozygous pale males being significantly longer than homozygous wild types, and with heterozygotes showing intermediate lengths (Figure 6b). Additionally, there does appear to be an advantage of having a pale father in terms of body length: Both males and females with pale fathers (treatments = WP and PP) had a larger mean body length than their counterparts which had wild‐type fathers (treatments = PW and WW), though this difference was only significant in males (*F*_3,195_ = 7.49, *p* = 0.007) but not females (*F*_3,191_ = 1.02, *p* = 0.313). As expected from previous work, there is sexual size dimorphism, with males being significantly smaller than females (*F*_1,388_ = 461.1, *p* < 0.001; see also Table 3 for the descriptive statistics). There was no effect of body length on female survival (*F*_1,191_ = 2.26, *p* = 0.134), but there was an effect of body length on the age male bugs lived to (*F*_1,192_ = 9.53, *p* = 0.002), with larger males living for longer.

**Table 3:** Descriptive statistics for Experiment 2 in terms of the mean body lengths of both males and females in each of the four experimental genotypes

Genotype	Female body length (mm)				Male body length (mm)			
	*N*	Mean	*SE*	Range	*N*	Mean	*SE*	Range
PP	46	11.66	0.06	10.5–12.45	47	10.87	0.05	9.90–11.55
PW	47	11.61	0.05	10.65–12.45	50	10.74	0.05	9.75–11.55
WP	50	11.65	0.06	10.35–12.45	50	10.84	0.05	10.05–11.40
WW	50	11.58	0.06	10.35–12.60	50	10.69	0.05	9.30–11.25

Finally, there was a significant positive association between the number of times a pair were observed mating and both the number of eggs laid (GLM: *F*_1,192_ = 9.42, *p* = 0.002), and the number of nymphs produced (*F*_1,192_ = 11.94, *p* < 0.001). This did not differ across treatments, either for the number of eggs laid (ANCOVA: number of times observed mating: *F*_1,186_ = 4.76, *p* = 0.030; treatment: *F*_3,186_ = 5.14, *p* = 0.002; interaction: *F*_3,186_ = 2.11, *p* = 0.101) or for the number of nymphs produced (number of times observed mating: *F*_1,186_ = 6.95, *p* = 0.009; treatment: *F*_3,186_ = 6.15, *p* < 0.001; interaction: *F*_3,186_ = 1.94, *p* = 0.125). Pairs which were observed to mate more often laid more eggs and produced more offspring than pairs which did not. Out of the 194 pairs, 176 were observed to mate at least once. Of the 18 pairs which were not observed to mate, 16 pairs laid eggs and seven pairs produced nymphs, so clearly our limited scan sampling did not catch all copulations. The number of times a female was observed mating was not associated with her body length (GLM: *F*_1,191_ = 0.42, *p* = 0.52), but larger males were observed to mate more often (*F*_1,192_ = 6.15, *p* = 0.014). There was no effect of female body length on the number of eggs laid either (*F*_1,191_ = 2.57, *p* = 0.111), or on the number of nymphs produced (*F*_1,191_ = 1.06, *p* = 0.306), and there was likewise no effect of male body length on the number of eggs his partner laid (*F*_1,192_ = 1.84, *p* = 0.177) or the number of nymphs sired (*F*_1,192_ = 2.19, *p* = 0.14).

## 4 DISCUSSION

Our results show that the pale mutation observed in our laboratory cultures of *Lygaeus simulans* is a recessive allele that is inherited in a strictly Mendelian fashion. Moreover, our experimental crosses suggest that the color locus is pleiotropically associated with female fecundity, female longevity, and to a smaller extent with male body size. Interestingly, the female fecundity data clearly show heterozygous advantage for both PW and WP heterozygotes. These data provide the tantalizing suggestion that color and life‐history traits are genetically associated, and given the undoubted polygenicity of the latter traits, perhaps we have uncovered a supergene complex underlying color patterns in *Lygaeus*.

Color patterns have been shown to be genetically correlated with life‐history traits in several species, for example body size in *Hypolimnas* butterflies (Gordon & Smith, 1998), body size, clutch size, and egg size in pygmy grasshoppers (Ahnesjö & Forsman, 2003; Forsman, 1999), and immune defense, clutch size, egg mass, and thermoregulatory capability in side‐blotched lizards (Sinervo & Svensson, 2002; Svensson, McAdam, & Sinervo, 2009). Therefore, it should not come as a surprise that color morph in *L. simulans* appears to be genetically associated with other life‐history traits.

These associations we have seen could be a result of the gene controlling color morph being pleiotropic and hence influencing the other traits directly. For example, in *Drosophila melanogaster*, the different polymorphisms of the genes *Dopa decarboxylase* (*Dcd*) and *Catecholamines up (Catsup)*, which are involved in the synthesis of dopamine, were associated with variation in life span, among other behavioral and morphological traits (Carbone et al., 2006; De Luca et al., 2003). This is likely because genes controlling hormonal pathways will affect many different aspects of an animal's physiology, because many different traits are affected by the same hormones.

Alternatively, instead of a single gene having a widespread pleiotropic effect, it could be that a cluster of tightly linked loci are segregating as a “supergene,” perhaps associated with a polymorphic chromosome inversion system, as has been suggested for *Heliconius numata* (Kronforst & Papa, 2015). This means that recombination in heterozygotes (or heterokarotypes, at the level of the whole inversion) is suppressed and so certain alleles at a color locus become associated with particular traits also influenced by alleles at loci in the inversion. This could well be the case in *L. simulans*, but further work needs to be carried out before any solid conclusions can be drawn on whether we have a supergene, or a single, highly pleiotropic gene. One further step will be to look at inter‐specific crosses with *L. equestris* (Evans et al., 2015), as we may predict reproductive incompatibilities to become associated with different karyotypes as they evolve quasi‐independently. Indeed, this system may also therefore provide a further test of the role of chromosome inversions in the evolution of reproductive isolation and speciation.

Our results do not just suggest a pleiotropic gene or a supergene, however. Our data also suggest a role for sexually antagonistic selection in the maintenance of color polymorphism in an aposematic species. This is because the pale mutant leads to a reduction in female fitness, in terms of reduced longevity (albeit in the laboratory, in the absence of predators), but a possible increase in male fitness, due to the increase in body size seen for pale males. A reduction on longevity could hamper female fitness as it reduces the time in which females can lay eggs and produce offspring, whereas males could benefit from increased body size as there is some evidence in *L. simulans* that larger males are more likely to mate than smaller males (V. Balfour, D. Black & D. M. Shuker, unpublished data). Such traits could be maintained by balancing selection, as has been suggested for side‐blotched lizards in which immunological defense is increased in orange morph males, but reduced in orange morph females (Svensson et al., 2009). However, the fitness consequences of male and female body size may be environment dependent. Increased body size may be associated with an increase in predation or parasitism, as has been suggested for individuals of the Sicily population of *L. equestris*. Individuals in this population are much smaller than their northern counterparts, which has been suggested to be due to the presence of predators in the southern range—in particular tachinid flies which preferentially predate on larger *L. equestris *females (Solbreck, Olsson, Anderson, & Förare, 1989).

We did not test here whether the pale mutants were more prone to predation than their aposematic wild‐type counterparts. Sillén‐Tullberg (1985a; 1985b) did find reduced fitness in her gray mutant fifth‐instar *L. equestris *nymphs, which were predated more readily than wild‐type nymphs under laboratory conditions. Therefore, it is possible that our pale mutants would likewise have reduced fitness in terms of increased mortality in the wild due to predation, though this remains to be tested. To the best of our knowledge, there have been no sightings of pale mutants in the wild, so if similar color mutants have arisen in the field, it could be that the recessive gene persists in wild populations in heterozygous individuals while homozygous recessives are kept at a minimum in the population due to predation. Alternatively, this mutation could solely be a laboratory phenomenon which has arisen independently in our laboratory. The discovery of a pale individual in the wild would prove otherwise.

Interestingly, female body size had no effect on fecundity. This goes against previous findings in this species and the sister species *L. equestris*; that is, bigger females do typically lay more eggs (in *L. equestris*: Shuker et al, 2006) and produce more offspring (in *L. simulans*: Dougherty & Shuker, 2016). However, here fecundity it is likely driven by age instead. Females which lived longer laid more eggs. There was no relationship between body size and age, so it might just be that the effect of body size on number of eggs laid was not strong enough over the course of the whole experiment, where we might have seen that effect if we would just looked at number of eggs laid in the first 2 weeks post‐mating, before a significant proportion of the females died. However, clearly heterozygosity is the strongest driver of fecundity in this study and should be the focus of future investigations.

To conclude, the discovery that color morph in *L. simulans* is controlled by a recessive allele inherited in a Mendelian fashion will be a useful tool for carrying out future experiments, as it can be used as a visible genetic marker. For example, it could be used to characterize sperm competition and other post‐copulatory mechanisms of sexual selection. For instance, this visible mutant will also allow us to test for the first time whether mating failure, previously shown to be a male associated phenotype (Greenway & Shuker, 2015; Greenway et al., 2017), is also a female associated phenotype, indicative of cryptic female choice for example; such work is currently underway. However, we note that for such experiments to be carried out, the observed heterozygous advantage in terms of fecundity must be taken into account, especially across multigenerational experiments.

## AUTHOR CONTRIBUTIONS

VLB and DMS conceived the project and designed the experiments. VLB and CA carried out the experimental work. LRD isolated and initially maintained the pale *L. simulans* mutant. VLB analyzed the data. VLB and DMS wrote the manuscript. All authors read and approved the manuscript.

## DATA ACCESSIBILITY

Our data have been archived in Dryad. https://doi.org/10.5061/dryad.56d7540.
